# A randomized, double-blinded trial to evaluate two therapeutic foods as adjunctive therapies in dogs with environmental and/or food-induced atopic dermatitis

**DOI:** 10.1093/jas/skag251

**Published:** 2026-08-11

**Authors:** Cassidy Yanes, Michelle LeRoy, David Senter, Laura A Motsinger, Morgan A Key

**Affiliations:** Veterinary Allergy and Dermatology Clinic, 11728 Quivira Road , Overland Park, KS 66210, United States; Veterinary Allergy and Dermatology Clinic, 11728 Quivira Road , Overland Park, KS 66210, United States; Veterinary Allergy and Dermatology Clinic, 11728 Quivira Road , Overland Park, KS 66210, United States; Hill’s Pet Nutrition Center, 1036 NE 43rd Street, Topeka, KS 66617, United States; Hill’s Pet Nutrition Center, 1036 NE 43rd Street, Topeka, KS 66617, United States

**Keywords:** allergies, atopic dermatitis, canine, nutrition, pruritus, skin lesions

## Abstract

This study evaluated changes in pruritus, skin lesions, and gastrointestinal signs (G) in dogs fed a positive Control food, Hill’s Prescription Diet d/d Potato & Venison (Hill’s Pet Nutrition, Topeka, KS, USA) compared to a Test food, Hill’s Prescription Diet Derm Complete (Hill’s Pet Nutrition, Topeka, KS, USA), over an 8-wk period. We hypothesized that feeding either food would result in improvements in skin lesions and pruritus when compared to baseline while maintaining positive GI and quality of life (QoL) outcomes, but that the Test food would yield superior improvement compared to the positive Control due to its specific immunomodulating components. Thirty-nine client-owned dogs with canine atopic dermatitis were randomly assigned to either the positive Control (*n* = 20) or Test (*n* = 19) food to feed strictly for 56 d. Veterinary dermatologists performed physical exams, recorded medication scores, and evaluated skin lesions using the Canine Atopic Dermatitis and Severity Index, 4th iteration (CADESI-4). Pruritus was evaluated by pet owners using a validated Pruritus Visual Analog Scale (PVAS). QoL, stool quality, coat quality, and eating enthusiasm were assessed via owner questionnaires. All other endpoints were analyzed using non-parametric methods. No significant differences were present between the Control and Test groups for any endpoints assessed. No differences in medication scores were observed over time for either the Control (*P *= 0.356) or Test (*P *= 0.304) groups. Both groups exhibited improvements in CADESI-4 over time (Control: *P *< 0.001; Test: *P *< 0.001). Owner-reported PVAS (Control: *P *= 1.00; Test: *P *= 1.00), stool quality (Control: *P *= 1.00; Test: *P *= 1.00), coat quality (Control: *P *= 1.00; Test: *P *= 0.908), and eating enthusiasm (Control: *P *= 0.427; Test: *P *= 0.215) remained stable throughout the study for both groups. Owners of dogs in the Control group had no significant changes (*P *= 0.518) in their QoL over time, whereas owners of dogs in the Test group had improvements (*P *= 0.022) in their QoL over time. No differences in pet QoL were observed over time for the Control (*P *= 0.309) or Test (*P *= 0.687) groups. These data suggest that feeding either the Test or Control foods results in notable improvements in skin lesions over time. Canine atopic dermatitis requires long-term management using a combination of medication and dietary modifications. These foods may play a positive role as an adjunctive therapy in multimodal management plans.

## Teaser

The present study demonstrates that feeding dogs specific therapeutic dermatologic foods improves skin lesions associated with canine atopic dermatitis over time, supporting their use as adjunctive therapy for allergy management in dogs.

## Introduction

Canine atopic dermatitis (cAD) is a common, chronic inflammatory skin disease characterized by a defective skin barrier that predisposes dogs to penetration by allergenic proteins, triggering abnormal inflammatory and allergic responses (Nuttall et al. 2019). Barrier dysfunction arises from abnormalities in epidermal composition, tight junctions, and filaggrin expression (Nuttall et al. 2019; Banovic 2025). This leads to increased transepidermal water loss and allergen sensitization (Nuttall et al. 2019; Banovic 2025). Once this occurs, cyclic outbreaks of inflammation and infections ensue (Marsella 2026). Genetics and environmental factors interact to influence the development and progression of cAD, with emerging evidence highlighting lifestyle, stress, diet, gut, and skin microbiome as areas of interest (Nuttall et al. 2019; Banovic 2025; Marsella 2026).

Diagnosis of cAD relies on a collection of compatible historical and clinical features (Olivry et al. 2010; Nuttall et al. 2019; Banovic 2025). Affected dogs typically have a history of seasonal or nonseasonal pruritus often accompanied by recurrent skin or ear infections (Olivry et al. 2010). Food-induced atopic dermatitis (AD) and environmental-induced AD can appear similar clinically (Hensel et al. 2015). Furthermore, adverse food reactions concurrent to AD have been documented to account for 20% to 30% or more of dogs (Bhagat et al. 2017). As such, classification requires a strict 8-wk elimination diet trial with a hydrolyzed or novel protein diet (Hensel et al. 2015). Food-induced AD is confirmed when there is evidence of a flare on provocation (Hensel et al. 2015). Proper identification of food allergens allows owners to avoid exposure as a way to manage this condition long term. It is equally important that other conditions are appropriately ruled out, as many inflammatory dermatoses can mimic cAD (Olivry et al. 2010; Hensel et al. 2015). Ectoparasites and skin infections with *Staphylococcus* spp. or *Malassezia* commonly complicate diagnosis (Olivry et al. 2010; Hensel et al. 2015). Once a diagnosis of cAD is made, targeted therapies such as allergen-specific immunotherapy (ASIT) and/or medications targeting inflammation and pruritus can be employed.

Management of cAD is often multimodal and targets various disease components to reduce inflammation, control pruritus, and improve quality of life (QoL) for pets and owners (Olivry et al. 2010; Nuttall et al. 2019). Therapeutic options include ASIT, topical therapies and systemic medications such as glucocorticoids, cyclosporine, JAK inhibitors (oclacitinib or ilunocitinib), monoclonal antibodies (lokivetmab), or antihistamines (diphenhydramine, cetirizine, etc.) (Olivry et al. 2010; Nuttall et al. 2019). Despite these advances, variable patient responses hinder complete resolution, while long-term medication use risks side effects, costs, and owner fatigue (Linek and Favrot et al. 2010; Cosgrove et al. 2015; Fennis et al. 2022; Banovic 2025; Marsella 2026).

Nutritional interventions have historically been recognized as effective adjunctive therapies (Glos et al. 2008). Studies have demonstrated reductions in pruritus and/or skin lesions in dogs fed specific dermatologic foods (Glos et al. 2008; Witzel-Rollins et al. 2019; Watson et al. 2022). Specific dietary approaches for supporting dogs with cAD include utilizing novel or hydrolyzed protein sources to minimize exposure to food allergens, as well as optimizing the balance of essential fatty acids to improve skin barrier function and reduce inflammation (Glos et al. 2008; Nuttall et al. 2019; Witzel-Rollins et al. 2019; Watson et al. 2022). However, evidence remains inconsistent; data on the efficacy of dermatologic foods commonly recommended in practice using real-world multimodal management plans remains limited, with few high-quality trials comparing veterinary therapeutic foods head-to-head.

Thus, the present study aimed to evaluate two different nutritional strategies. The positive Control food utilizes a single uncommon animal protein source (venison) to avoid common allergens, functioning as a standard avoidance food. Conversely, the Test food utilizes a single, intact animal protein (egg) and is fortified with flavonoid-containing ingredients and higher levels of total Omega-3 fatty acids (1.73% vs. 0.94%) to help normalize immune responses.

The purpose of this study was to evaluate changes in pruritus, skin lesions, and gastrointestinal (GI) signs in dogs with cAD fed one of two veterinary foods marketed for AD in conjunction with standard therapies. Additional objectives included assessing changes in concomitant medication needs, allergy-related behaviors, pet-perceived QoL, and owner QoL in relation to disease burden. We hypothesized that feeding the positive Control food, Hill’s Prescription Diet d/d Potato & Venison (Hill’s Pet Nutrition, Topeka, KS, USA) or the Test food, Hill’s Prescription Diet Derm Complete (Hill’s Pet Nutrition, Topeka, KS, USA), as an adjunctive therapy would significantly improve skin lesions and pruritus, reduce or maintain the need for concomitant allergy medications, and maintain positive gastrointestinal and quality-of-life outcomes in dogs with cAD. Furthermore, we hypothesized that the Test food would demonstrate superior efficacy over the positive Control food due to its higher levels of Omega-3 fatty acids and fortified flavonoid-containing ingredients.

## Materials and methods

### Animal use and informed consent

All procedures were directed by a protocol (CP1143a) approved by the Institutional Animal Care and Use Committee of Hill’s Pet Nutrition, Inc., in Topeka, KS, USA and were in accordance with the Hill’s Global Animal Welfare Policy. Signed informed consent was obtained for all clients entering their dogs into the study.

### Animals

All of the study participants were enrolled at a private dermatology practice, and evaluations were performed by a board-certified dermatologist or dermatology resident at each visit. Qualifying dogs had been diagnosed with cAD by a veterinarian based on Favrot’s criteria and were eligible for enrollment if they had non-seasonal pruritus (Hensel et al. 2015). In addition to the aforementioned criteria, one of the following added criteria needed to be met to allow for inclusion in the study: pruritus visual analog scale (PVAS) score ≥ 2 or Canine Atopic Dermatitis Extent and Severity Index, 4th iteration (CADESI-4) score ≥ 10. Prior 8-wk elimination diet trials with a hydrolyzed or novel protein diet and/or intradermal or serum allergy test were allowed but not required for enrollment. Dogs with superficial pyoderma and/or malassezia dermatitis were allowed enrollment if the dog had a PVAS ≥ 2 and/or CADESI-4 ≥ 10, concluding appropriate antibiotic or antifungal treatment, and improvement was confirmed via cytologic evaluation (Loeffler et al. 2025). Dogs included in the study were ≥1 yr of age. All dogs were required to be up to date with external antiparasitic treatments that excluded flea and mite infections at the time of enrollment and throughout the study period. Signalment of dogs can be found in Table 1.

**Table 1:** Dog characteristics at study baseline prior to transitioning on to the Control food or Test food for the 56-d study period.

Characteristic	Control group^1^	Test group^1^	*P*-value
**Animals, *n***	20	19	-
**Intact male**	1	0	0.1497
**Neutered male**	7	12	
**Spayed female**	12	7	
**Age, y**	5.65 (0.63)	4.79 (0.60)	0.3315
**Body weight, kg**	21.91	27.45 (2.93)	0.1849
**Breed, *n***	-	-	-
**American Pit Bull Terrier**	0	2	-
**American Pit Bull Terrier cross**	0	2	-
**American Staffordshire Terrier**	0	1	-
**Australian cattle cross**	1	0	-
**Australian Shepherd**	1	0	-
**Beagle cross**	1	0	-
**Boxer**	1	1	-
**Boxer cross**	0	1	-
**Bulldog**	1	1	-
**Chihuahua**	1	0	-
**Chihuahua cross**	1	2	-
**Crossbreed**	0	1	-
**French Bulldog**	2	1	-
**German Shepherd cross**	0	1	-
**Golden Retriever**	0	1	-
**Golden Retriever cross**	3	1	-
**Labrador Retriever**	1	0	-
**Labrador Retriever cross**	1	2	
**Lhasa Apso cross**	0	1	-
**Newfoundland**	0	1	-
**Pekingese cross**	1	0	-
**Poodle cross**	2	0	-
**Pug cross**	1	0	-
**Siberian Husky**	1	0	-
**Yorkshire Terrier cross**	1	0	-

1 Data are n or means (SE).

Concomitant dermatology-related medications were allowed throughout the study to ensure an appropriate QoL for all pets. Medications were allowed so long as these had been unchanged within 1 mo prior to enrollment. A medication scoring system (Fischer et al. 2018; Watson et al. 2022) (Table 2) was utilized to track the doses and frequencies of medications needed throughout the 8-wk study period.

**Table 2:** Medication scoring system (Fischer et al. 2018; Watson et al. 2022) used to grade medication consumption by dogs on d −7, 28, and 56.

Medication and dose range	Score
**No current medications**	0
**Medicated shampoo**	5
**Topical ear medication**	5
**Other topical therapies**	5
**Antihistamines**	10
**Frequent antibiotics (>21 d)**	20
**Less frequent antibiotics (<21 d)**	10
**Glucocorticoids (≥1 mg/kg/d)**	40
**Glucocorticoids (0.5–1 mg/kg/d)**	30
**Glucocorticoids (0.2–0.5 mg/kg/d)**	20
**Glucocorticoids (≤0.2 mg/kg/d)**	10
**Cyclosporine (5 mg/kg) or ∼2.5 mg/kg + ∼5 mg/kg ketoconazole**	
**Once per day dosing of cyclosporine**	30
**Every other day dosing of cyclosporine**	20
**Every 3-d dosing of cyclosporine**	10
**Every 4-d dosing of cyclosporine**	5
**Oclacitinib twice per day dosing**	40
**Oclacitinib once per day dosing**	30
**Oclacitinib every other day dosing**	20
**Ilunocitinib once per day dosing**	30
**Ilunocitinib every other day dosing**	20
**Lokivetmab administered every 4 wk**	20
**Lokivetmab administered >4 wk**	10

Dogs were excluded from the study if they were on ASIT for less than 12 mo. Dogs were not allowed for enrollment if they were pregnant or lactating or had other chronic conditions that could affect study outcomes, such as a history of chronic GI disease. Other disqualifications included an exclusively outdoor lifestyle, fractious behavior exhibited in the clinic, or if they had access to other pets’ food in the household and would not be able to feed the assigned food strictly. Dogs were not allowed to continue in the study if they experienced anorexia for 72 h. Serious concurrent medical conditions omitted pets from enrollment, specifically if they needed to be administered a different therapeutic food that would preclude them from being recommended either of the study foods.

### Study design and dietary intervention

The present study was a randomized, double-blinded trial that took place over 56 d. Dogs enrolled in the study were randomly assigned to one of two study groups identified as the Control group or Test group. The Control and Test foods were provided directly by Hill’s Pet Nutrition. Brand labeling was omitted, and all food was supplied in identical, white packaging with a colored label (red or yellow) for differentiation of the Control and Test food. Red was the color assigned to the Control food, Hill’s Prescription Diet d/d Potato & Venison (Hill’s Pet Nutrition, Topeka, KS, USA), and yellow was the color assigned to the Test food, Hill’s Prescription Diet Derm Complete (Hill’s Pet Nutrition, Topeka, KS, USA).

The rationale of this study design was to compare an established, standard-of-care nutritional approach (the positive Control) against a novel, multimodal nutritional strategy (Test food) to evaluate their comparative efficacy in managing cAD. The positive Control food utilizes a single, non-traditional intact animal protein (venison) and potato as the primary carbohydrate source. The positive Control food is formulated to minimize patient exposure to common dietary allergens; historically, beef, dairy products, and wheat account for approximately 69% of reported adverse food reactions in dogs (Bhagat et al. 2017).

Conversely, the Test food contains egg as its primary intact animal protein, supplemented with a plant-based rice protein concentrate. Egg was selected because it is an excellent quality protein source, as evidenced by its average Digestible Indispensable Amino Score of above 100 (Herreman et al. 2020), and it is not commonly associated with canine adverse food reactions (CAFR), representing only about 4% of documented food allergy cases in dogs, and it contains specific components demonstrated to possess innate immunomodulating effects (Mueller et al. 2016). During production of these foods, a complete equipment breakdown, cleaning, sanitation, and inspection are performed between proteins to help minimize cross-contamination.

Beyond protein selection, the Test food was formulated with distinct differences in its fatty acid and antioxidant profiles to actively target skin barrier dysfunction. The Test food contains a higher concentration of total Omega-3 fatty acids (1.73% in the Test food compared to 0.94% in the Control food on a dry matter basis). Supplemental Omega-3 fatty acids have been shown to significantly reduce pro-inflammatory markers, including IL-6, TNF-alpha, and C-reactive protein, in dogs with AD (Kareem and Tawfeeq 2025). Additionally, the Test food includes a proprietary nutrient bundle selected based on naturally occurring specific flavan-3-ol compounds which have been shown to modulate the immune system directly by inhibiting the degranulation of mast cells (Sommerhoff et al. 1990; Singh et al. 2011; MacLeay et al. 2016; Gross et al. 2018).

The Test food delivers a higher concentration of Vitamin E (1,189.1 IU/kg) compared to the Control (964.5 IU/kg), but both foods exceed the Association of American Feed Control Officials (AAFCO) adult maintenance minimum recommendation of 50 IU/kg on a dry matter basis (AAFCO 2026). Additionally, although no AAFCO minimum maintenance recommendation has been established, research has shown that a food enriched with Vitamin C at or exceeding 98 ppm can help improve skin health and reduce inflammation (de Santiago et al. 2021). The Test food contains a higher concentration of Vitamin C (209.7 ppm) compared to the Control food (150.3 ppm). This elevated antioxidant profile is designed to offer enhanced protection against oxidative stress and the subsequent lipid peroxidation commonly observed in the stratum corneum of dogs with dermatologic disease (Jewell et al. 2002). Nutritional analysis was conducted by Eurofins Scientific, Inc. (Des Moines, IA, USA) and Covance Laboratories (Princeton, New Jersey, USA) using official methods of analysis published by AOAC International (2019). Pet owners and all personnel employed by the private dermatology practice were blinded to the study foods. The nutrient composition and list of ingredients for each food can be found in Table 3. Daily energy requirements for enrollees were calculated by multiplying the resting energy requirement (RER) by the activity factor of 1.6. RER for dogs was determined as follows: RER (kcal/d) = 70 × BW_kg_^0.75. A 7-d transition to the Control or Test food was required to allow smooth integration. Day 1 was defined as the start day for the 8-wk study period, where pets were fully transitioned to their assigned food 7 d after their enrollment and examination in clinic. Foods outside of the assigned study food, including treats, wet foods, and flavored supplements, were not permitted during the study. Flea and tick products with animal protein flavoring were continued for patients receiving this form of prevention throughout the study. Approved treats and pill pockets, including Potato Pleasers dog treats and Potato Wrap It (Serenegy Dog Treats, Beaverton, Oregon, USA), were allowed and provided to owners upon request. This was a neutral treat item with limited ingredients consisting of potato starch, potato flour, unsulfured molasses, olive oil, baking soda, and mixed tocopherols.

**Table 3:** Analyzed^3^ nutrient composition of the Control and Test foods.

Nutrient^1^	Control group^4^	Test group^5^
**ME (calculated), kcal/kg^1^^,^^6^**	3,917	4,179
**Crude protein, %**	18.7	16.8
**Protein source**	Venison and potato	Dried whole eggs and rice protein concentrate
**Crude fat, %**	14.9	17.7
**NFE (calculated), %^1^^,^^7^**	57.0	59.7
**Carbohydrate source**	Potatoes and potato starch	Brewers’ rice
**Crude fiber, %**	3.6	1.3
**Calcium, %**	0.99	0.62
**Phosphorus, %**	0.68	0.63
**Potassium, %**	1.05	0.93
**Sodium, %**	0.40	0.23
**Magnesium, %**	0.06	0.13
**Vitamin C, ppm**	150.3	209.7
**Vitamin E, IU/kg**	964.5	1,189.1
**DHA, %^1^**	0.14	0.27
**EPA, %** ^1^	0.23	0.40
**Total Omega-3, %**	0.94	1.73
**Total Omega-6, %**	3.65	3.95
**Omega 6:3 ratio**	3.9:1	2.3:1

^1^DHA, docosahexaenoic acid; EPA, eicosapentaenoic acid; ME, metabolizable energy; NFE, nitrogen-free extract.

^2^All nutrients are presented on a dry matter basis.

3 Nutritional analysis was conducted by Eurofins Scientific, Inc. (Des Moines, IA, USA) and Covance Laboratories (Princeton, New Jersey, USA) using official methods of analysis published by AOAC International (2019).

4 The control food, Hill’s Prescription Diet d/d Potato & Venison included the following ingredients: Potatoes, Potato Starch, Venison, Potato Protein, Soybean Oil, Coconut Oil, Powdered Cellulose, Pork Liver Flavor, Dicalcium Phosphate, Lactic Acid, Fish Oil, Potassium Chloride, Glyceryl Monostearate, Calcium Carbonate, Iodized Salt, Choline Chloride, vitamins (Vitamin E Supplement, L-Ascorbyl-2-Polyphosphate (source of Vitamin C), Niacin Supplement, Thiamine Mononitrate, Calcium Pantothenate, Pyridoxine Hydrochloride, Vitamin A Supplement, Riboflavin Supplement, Biotin, Vitamin B12 Supplement, Folic Acid, Vitamin D3 Supplement), DL-Methionine, Taurine, minerals (Ferrous Sulfate, Zinc Oxide, Copper Sulfate, Manganous Oxide, Calcium Iodate, Sodium Selenite), Mixed Tocopherols for freshness, Magnesium Oxide, Natural Flavors, Beta-Carotene.

5 The Test food, Hill’s Prescription Diet Derm Complete included the following ingredients: Brown Rice, Brewers Rice, Egg Product, Rice Protein Concentrate, Soybean Oil, Flaxseed, Hydrolyzed Chicken Flavor, Dried Beet Pulp, Fish Oil, Coconut Oil, Lactic Acid, Dicalcium Phosphate, Potassium Chloride, Calcium Carbonate, Lipoic Acid, Iodized Salt, vitamins (Vitamin E Supplement, L-Ascorbyl-2-Polyphosphate [source of Vitamin C], Niacin Supplement, Thiamine Mononitrate, Vitamin A Supplement, Calcium Pantothenate, Riboflavin Supplement, Biotin, Vitamin B12 Supplement, Pyridoxine Hydrochloride, Folic Acid, Vitamin D3 Supplement), Green Peas, Taurine, Apples, Cranberries, Choline Chloride, Carrots, DL-Methionine, Natural Flavors, minerals (Ferrous Sulfate, Zinc Oxide, Copper Sulfate, Manganous Oxide, Calcium Iodate, Sodium Selenite), Mixed Tocopherols for freshness, Broccoli, Beta-Carotene.

6 ME was calculated based on modified Atwater values.

7 NFE was calculated as follows: % NFE = 100% – (% ether extract + % crude protein + % ash + % crude fiber).

### Study assessments

Owners were instructed to return for in-person evaluations at different timepoints at the beginning, middle, and end of the study outlined in Table 4. At each in-clinic visit on d −7, 28, and 56, a physical exam was performed by a board-certified veterinary dermatologist or dermatology resident, and body weight and body fat index were recorded to ensure that dogs maintained ideal body weight. The dog’s overall skin condition was evaluated by a board-certified dermatologist or dermatology resident using the validated CADESI-4 to measure erythema, lichenification, and alopecia or excoriations on 20 body sites on d −7, 14, 28, and 56 (Olivry et al. 2014). CADESI-4 score benchmarks for mild, moderate, and severe disease were ≥ 10, ≥ 35, and ≥ 60, respectively.

**Table 4:** Timepoints when physical exams, medication scores, CADESI-4, PVAS, and client questionnaires (assessing stool sore, coat quality, eating enthusiasm, and QoL) were collected throughout the 8-wk study period.

Datapoints and frequency of collection						
	Day −7^1^	Day 1^2^	Day 14	Day 28	Day 42	Day 56
**Physical exam in clinic**	X			X		X
**Medication score**	X			X		X
**CADESI-4**	X		X	X		X
**PVAS**	X		X	X	X	X
**Stool scores**		X	X	X	X	X
**Coat quality**		X		X		X
**Eating enthusiasm**		X		X		X
**QoL questionnaires**		X		X		X

1 Day **−**7 served as the baseline measurement for body weight, medication score, CADESI-4, and PVAS.

2 Day 1 served as the baseline measurement for stool scores, coat quality, eating enthusiasm, and QoL questionnaires.

Simultaneous use of antipruritic and anti-inflammatory medications was assessed by a board-certified dermatologist or dermatology resident on d −7, 28, and 56. Doses and frequency of administration were recorded at each visit using a medication scoring system published previously (Fischer et al. 2018; Watson et al. 2022). Although this medication scoring system is not a universally standardized tool, it comprehensively accounts for common anti-pruritic therapies needed throughout the study period to maintain each patient’s condition. Each patient was assigned a cumulative medication score based on the chart described in Table 2. Importantly, no additional medical therapies that directly impacted the inflammatory or pruritic response were utilized outside of this provided scoring system. Medication scores were determined by summing daily scores and then taking the monthly totals divided by the number of days in the month to provide a daily average.

Owners assessed pruritus using a validated PVAS ranging from 0 (normal dog—I don’t think itching is a problem) to 10 (extremely severe itching/almost continuous) (Rybnícek et al. 2009) on d −7, 14, 28, 42, and 56. Owners also assessed stool quality, coat quality, eating enthusiasm, and QoL. All owner assessments were completed via online questionnaires. Stool quality was assessed using a six-point scale with an illustrated picture for classification, where a grade 1 indicated liquid stool and a grade 6 indicated firm stools (Figure S1, see online supplementary material for a color version of this figure). Coat quality was assessed using a validated scoring system evaluating six features, including alopecia, glossiness, greasiness, softness, scaliness, and overall coat quality (Devriendt et al. 2021). A numerical value was assigned to categorize the absence or presence of each condition or its severity using a 5-point scale (1 = normal, 5 = poor) (Devriendt et al. 2021). Eating enthusiasm, evaluated by an emoji scale ranging from 1 (least enthusiasm to eat) through 7 (most enthusiasm to eat). QoL was assessed via three sets of questions focused on the pet, pet parents, and treatments (Linek and Favrot 2010). Questions were scored from 0–4, where 4 was ideal such that higher QoL scores are more favorable. QoL score ranges for the pet, pet parents, and treatment question sets were 0–56, 0–64, and 0–24, respectively. Stool scores were recorded on d 1, 14, 28, 42, and 56. Coat quality, eating enthusiasm, and QoL questionnaires were submitted on d 1, 28, and 56.

### Statistical analysis

Body weight is a continuous measurement and is the only endpoint for which a parametric linear mixed model was utilized. The model included Group (Control vs. Test), Day (−7, 28, 56), and Group × Day interaction as fixed effects, with a random intercept per subject to account for repeated measurements.

The primary justification for non-parametric methods was the ordinal measurement level of all endpoints other than body weight. Shapiro-Wilk normality tests applied to pooled scores across groups and time points provided additional empirical support for this decision. To assess the between-diet effects, the Mann-Whitney U test (Wilcoxon rank-sum test) compared the two diet groups at each time point for each endpoint. This test assesses whether one group’s scores tend to be systematically higher or lower than the others without assuming normality or equal variances. Effect size: Cliff’s delta (d), ranging from −1 to 1, where 0 indicates no tendency for one group to exceed the other. The Friedman test (nonparametric repeated-measures ANOVA) assessed whether scores changed significantly across all available time points within each group. The Wilcoxon signed-rank test compared paired observations at each follow-up time point against baseline within each group. Effect size: rank-biserial correlation (r), computed as (positive ranks—negative ranks)/total non-zero differences. Multiple comparisons were performed within each endpoint. Two methods were applied to correct for multiplicity: the Holm-Bonferroni correction, which controls the family-wise error rate (FWER) and is the primary correction used for significance decisions for data analyzed in the present study, and the Benjamini-Hochberg (BH) correction, which controls the false discovery rate (FDR). Corrections were applied within each endpoint separately (e.g. all CADESI-4 tests corrected together, all PVAS tests together).

All statistical analyses were performed using Python (version 3.12). Effects were considered significant when *P *≤ 0.05.

## Results

### Study population

Forty-two dogs were enrolled in the study, and 39 completed all the study requirements to be considered for analysis, as shown in Table 1. Nineteen dogs (10 Control group; 9 Test group) that completed the study were new patients and enrolled upon their first visit in the clinic. In these cases, medical therapies were unchanged and continued throughout the 8-wk study period with no adjustments or interventions made by a specialist. The remainder of the 22 patients that were enrolled and completed the study were established clients that had been seen by a veterinary dermatologist and/or dermatology resident for multiple months or beyond 1 yr. Patients were enrolled between the months of June and November, with completion achieved for the final group by January. The strict inclusion criterion requiring all patients to exhibit perennial, non-seasonal pruritus was essential to effectively rule out any potential seasonal environmental influences during the study period. Three dogs were not considered for final analysis due to the development of neoplastic condition after enrollment, inability to feed the test food strictly because of anorexia, and incomplete client questionnaires. There were no differences in age (*P *= 0.332) or gender (*P *= 0.150) between the different groups at baseline. The average age of dogs enrolled in the Control group (*n* = 20) was 5.65 yr, and 4.79 yr for dogs in the Test group (*n* = 19). The Control group consisted of 1 (5%) intact male, 7 (35%) neutered males, and 12 (60%) spayed females. The Test group consisted of 12 (63.2%) neutered male dogs and 7 (36%) spayed female dogs.

Of the 42 dogs initially enrolled, 15 had previously completed an extensive 8-wk food elimination trial. Thirteen of these dogs had a food allergy definitively ruled out by the elimination trial. Two dogs had a confirmed food allergy (one dog in each group). Both dogs, however, suffered from food allergies and environmentally induced cAD as confirmed with intradermal skin testing and/or partial improvement in pruritus or skin lesions with the dietary trial and worsening observed upon re-challenge of their previous diets. At enrollment, both patients were already being managed on foods with hydrolyzed protein food (hydrolyzed chicken, *n* = 1; hydrolyzed soy, *n* = 1) and had controlled food allergies at baseline prior to transitioning to the study foods. Importantly, no pets in the study had undergone any dietary changes for a 3-to-6-mo period leading up to study enrollment. This strict dietary stability ensures that baseline values accurately reflect their maintained PVAS, CADESI-4, and medication needs prior to intervention with the Control or Test foods. Twenty-five dogs had an intradermal allergy test performed consisting of 14 dogs in the control group and eleven dogs in the test group. One dog in the control group had been serum allergy tested. All dogs that were allergy tested had significant positives to nonseasonal and seasonal allergens. Both foods were readily accepted and well tolerated during the 8-wk study period. Adverse events included recurrence of pyoderma in one patient fed the Control food at the end of the study period, one instance of vomiting in the Control group, three instances of vomiting in the Test group, one dog that refused to eat in the Control group, one staph infection in the Test group, and one instance of diarrhea in the Test group.

### Body weight

The Group × Day interaction was non-significant at both d 28 (*P *= 0.80) and d 56 (*P *= 0.78), indicating that the rate of body weight change did not differ between the Control and Test diet groups. Body weight results are displayed in Table 5.

**Table 5:** Descriptive statistics for body weight throughout the study for dogs fed the positive Control food and the Test food.

Group	Day	*n*	Median^1^	Q1	Q3	Min	Max
**Control**	−7	20	23.85	11.9	30.95	4.7	42.6
**Control**	28	20	24.25	11.675	30.75	5	44.9
**Control**	56	20	24.25	11.525	32	4.7	47.5
**Test**	−7	19	29.3	23.8	34.85	3.8	47.3
**Test**	28	17	30.6	22.4	34.5	4.1	50.4
**Test**	56	19	32.1	24.6	35.1	4.1	50.4

1 Body weight is represented in kilograms.

### Medication scores

**Figure 1:**
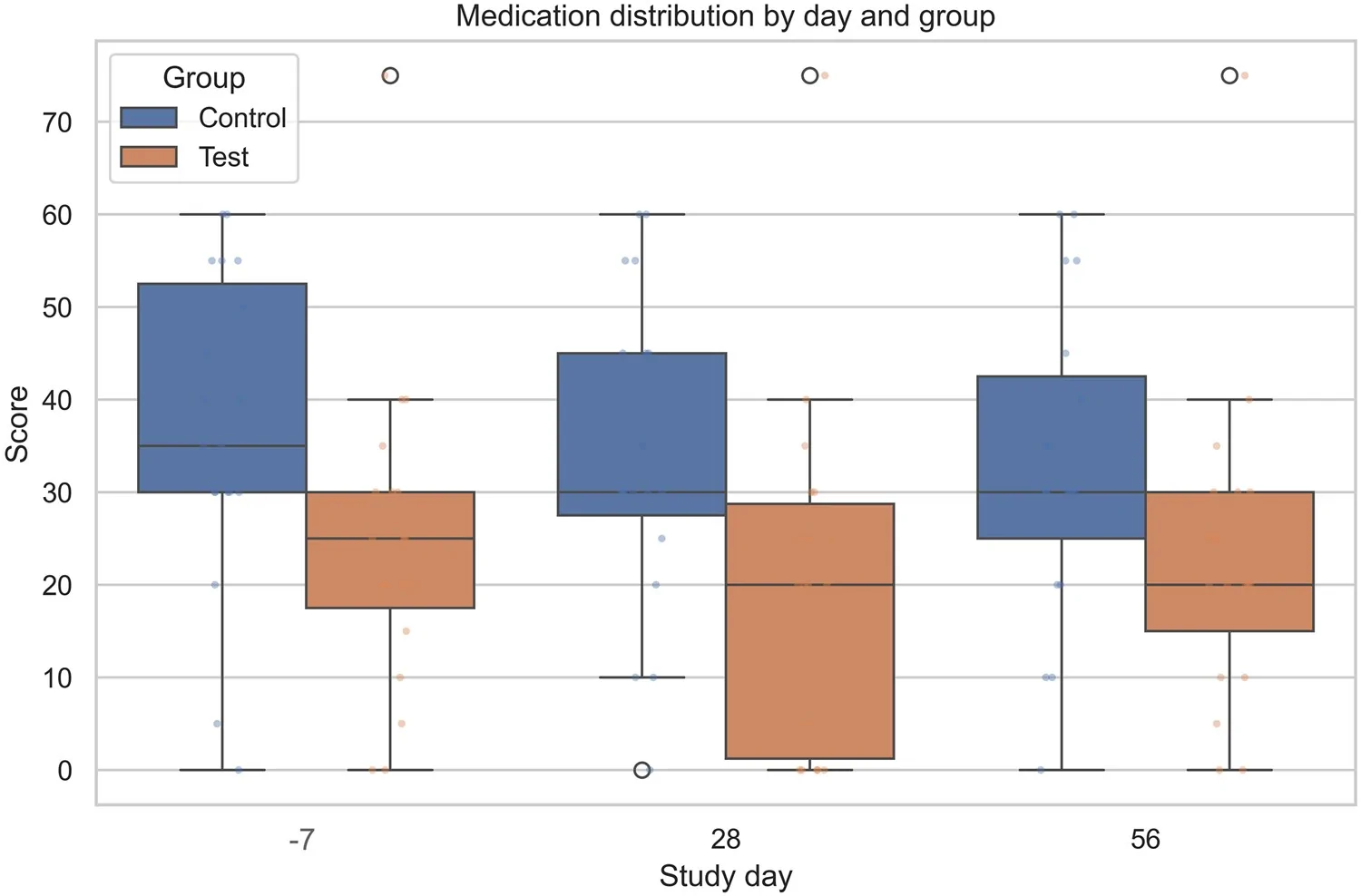
Medication scores determined by summing daily scores and then taking the monthly totals divided by the number of days in the month to provide a daily average for dogs fed the Control food or Test food.

No significant differences in medication scores were observed between the Control and Test groups at d −7 (*P *= 0.087), 28 (*P *= 0.104), or 56 (*P *= 0.224). Median medication scores at d −7, 28, and 56 were 35, 30, and 30 for the Control group, and 25, 20, and 20 for the Test group, respectively (Table 6, Figure 1, and Table S1). When evaluated over time, medication scores were maintained (Control: *P *= 0.356; Test: *P *= 0.304) in both groups (Table S1), Concurrent medical therapies such as JAK inhibitors, cyclosporine, glucocorticoids and/or lokivetmab had remained consistent for dogs enrolled in the study ranging from multiple months to over 1 yr prior to enrollment.

**Table 6:** Descriptive statistics for medication scores throughout the study for dogs fed the positive Control food and the Test food.

Group	Day	*n*	Median^1^	Q1	Q3	Min	Max
**Control**	−7	19	35	30	52.5	0	60
**Control**	28	19	30	27.5	45	0	60
**Control**	56	19	30	25	42.5	0	60
**Test**	−7	19	25	17.5	30	0	75
**Test**	28	18	20	1.25	28.75	0	75
**Test**	56	19	20	15	30	0	75

1 Medication scores were determined by summing daily scores and then taking the monthly totals divided by the number of days in the month to provide a daily average.

### CADESI-4

**Figure 2:**
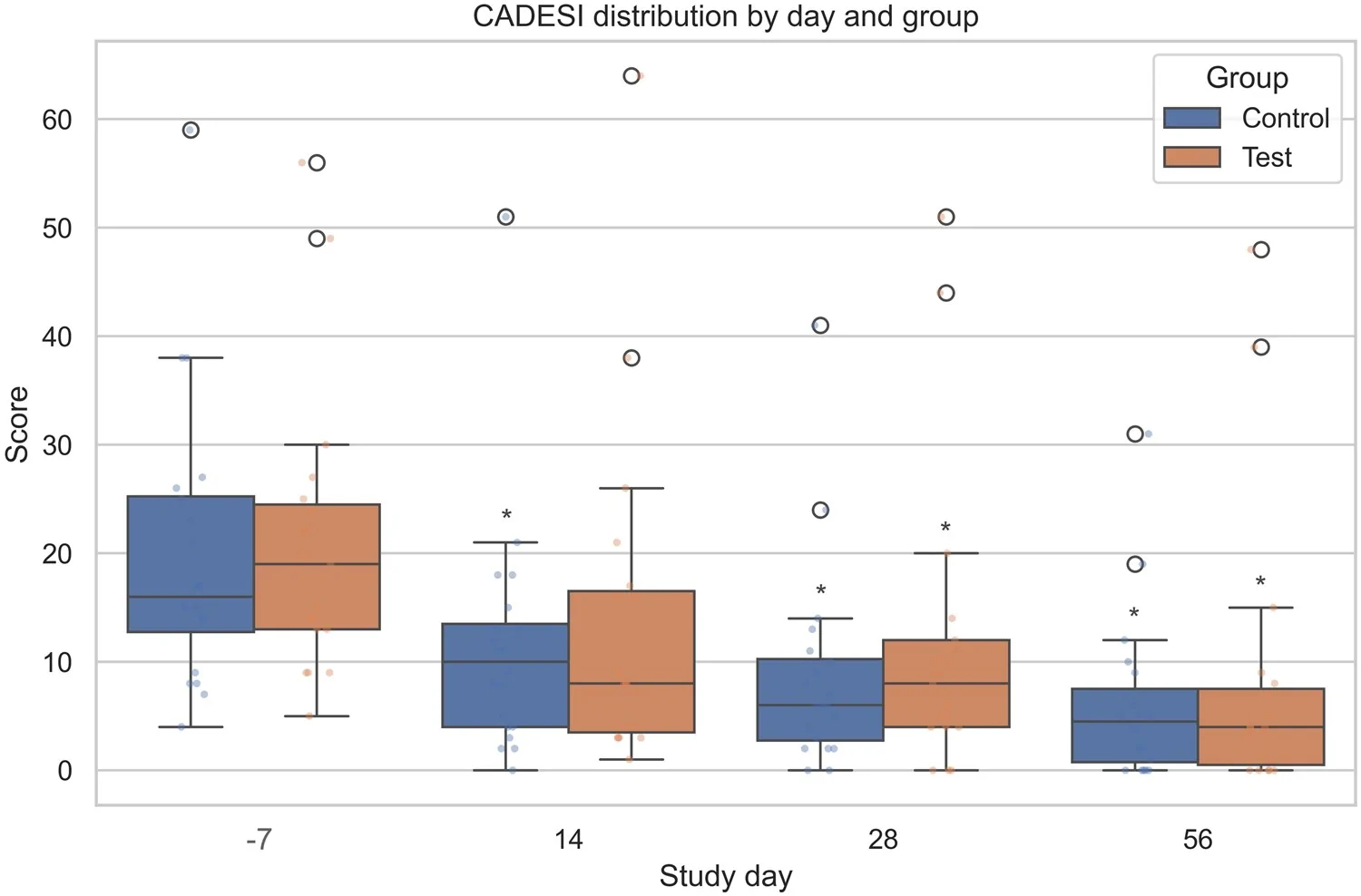
CADESI-4 scores assessed by veterinarians throughout the study duration for dogs fed the positive Control food or Test food. Benchmarks for mild, moderate, and severe disease are ≥10, ≥35, and ≥60, respectively. *P ≤ 0.05 compared to baseline.

No differences in CADESI-4 were present between the groups at d −7 (*P *= 1.00), 14 (*P *= 1.00), 28 (*P *= 1.00), or 56 (*P *= 1.00). Both the Control and Test groups demonstrated improvements over time in CADESI-4 (Control *P *< 0.001; Test *P *< 0.001; Table S1). Specifically, when compared to d −7, the Control group exhibited decreased CADESI-4 scores on d 14 (*P *= 0.001), 28 (*P *< 0.001), and 56 (*P *< 0.001). The Test group demonstrated significant improvements on d 28 (*P *= 0.002) and 56 (*P *< 0.001) when compared to baseline. No differences (*P *= 0.102) in CADESI-4 scores were observed in the Test group on d 14, when compared to baseline. The median baseline CADESI-4 scores for the Control and Test groups were 16 and 19, respectively, initially placing both groups in the mild disease category (≥ 10). By d 56, the median scores dropped to 4.5 and 4 for the Control and Test groups, respectively, which falls below the threshold for mild disease. Comparison to baseline CADESI-4 results is displayed in Table 7, Figure 2, and Table S2.

**Table 7:** Descriptive statistics for CADESI-4 scores throughout the study for dogs fed the positive Control food and the Test food.

Group	Day	*n*	Median^1^	Q1	Q3	Min	Max
**Control**	−7	20	16	12.75	25.25	4	59
**Control**	14	20	10	4	13.5	0	51
**Control**	28	20	6	2.75	10.25	0	41
**Control**	56	20	4.5	0.75	7.5	0	31
**Test**	−7	19	19	13	24.5	5	56
**Test**	14	19	8	3.5	16.5	1	64
**Test**	28	17	8	4	12	0	51
**Test**	56	19	4	0.5	7.5	0	48

1 CADESI-4 scores are based on the evaluation of three lesion types at 20 body sites. Lesions were assessed by veterinary dermatologists and measured on a scale of 0 (none) to 3 (severe), with 180 being the maximum total score. Benchmarks for mild, moderate, and severe disease were ≥10, ≥35, and ≥60, respectively.

### PVAS

**Figure 3:**
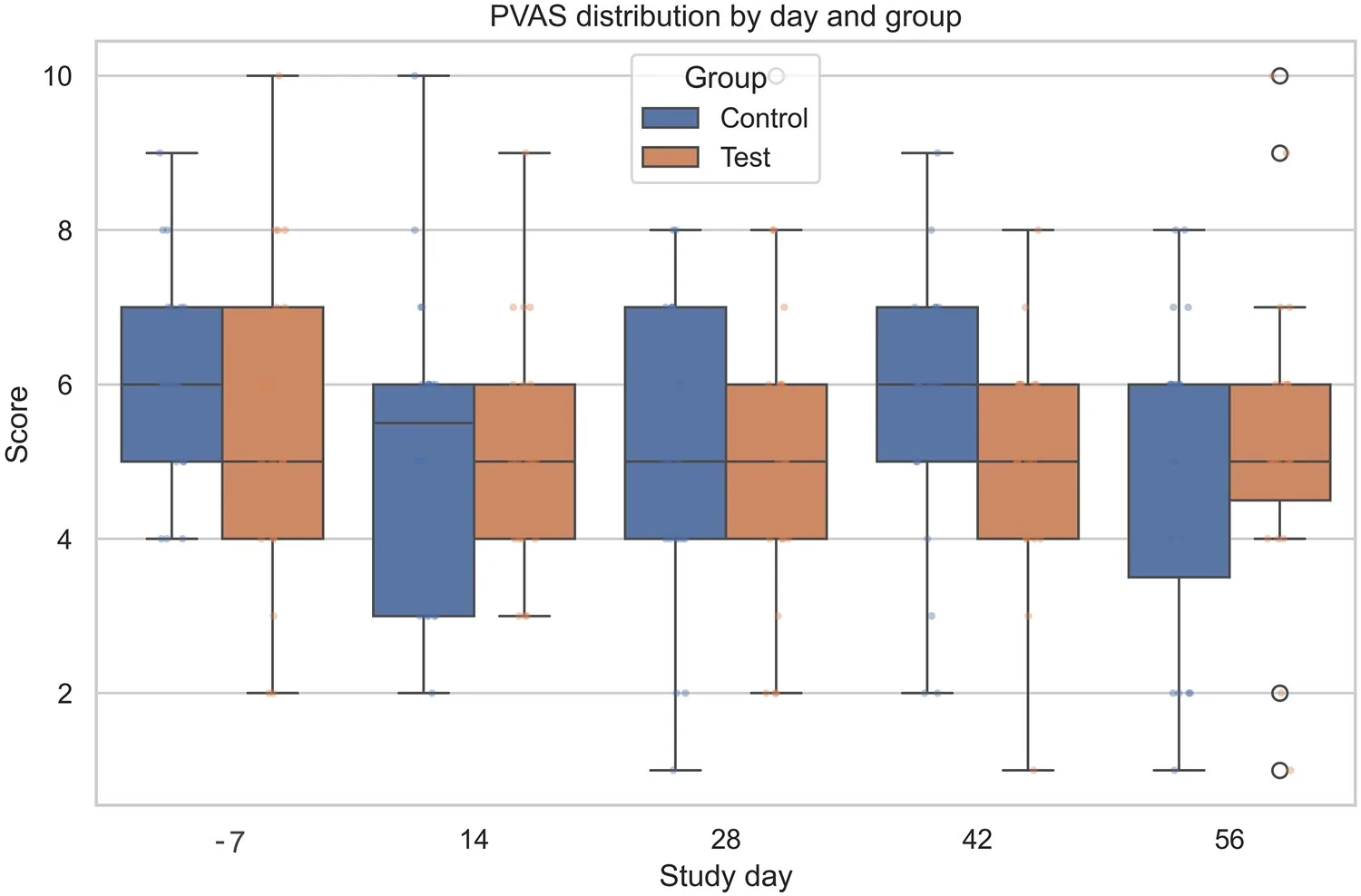
PVAS scores (0 = no pruritus, 10 = severe pruritus) assessed by pet owners throughout the study duration for dogs fed the positive Control food or Test food or Control. Severity is categorized as mild ≥2.0, moderate ≥5.0, and severe ≥8.0.

No differences in PVAS were observed between the Control and Test groups at d −7 (*P *= 1.00), 14 (*P *= 1.00), 28 (*P *= 1.00), 42 (*P *= 1.00), or 56 (*P *= 1.00). Additionally, PVAS was maintained (Control: *P *= 1.00; Test: *P *= 1.00) over time in both groups (Table 8, Figure 3, and Tables S1 and S2).

**Table 8:** Descriptive statistics for PVAS scores throughout the study for dogs fed the positive Control food and the Test food.

Group	Day	*n*	Median^1^	Q1	Q3	Min	Max
**Control**	−7	20	6	5	7	4	9
**Control**	14	20	5.5	3	6	2	10
**Control**	28	19	5	4	7	1	8
**Control**	42	18	6	5	7	2	9
**Control**	56	20	6	3.5	6	1	8
**Test**	−7	19	5	4	7	2	10
**Test**	14	19	5	4	6	3	9
**Test**	28	19	5	4	6	2	10
**Test**	42	18	5	4	6	1	8
**Test**	56	19	5	4.5	6	1	10

1 PVAS scores were assessed by owners on a 0–10 scale (0 = no pruritus, 10 = severe pruritus).

### Stool quality

**Figure 4:**
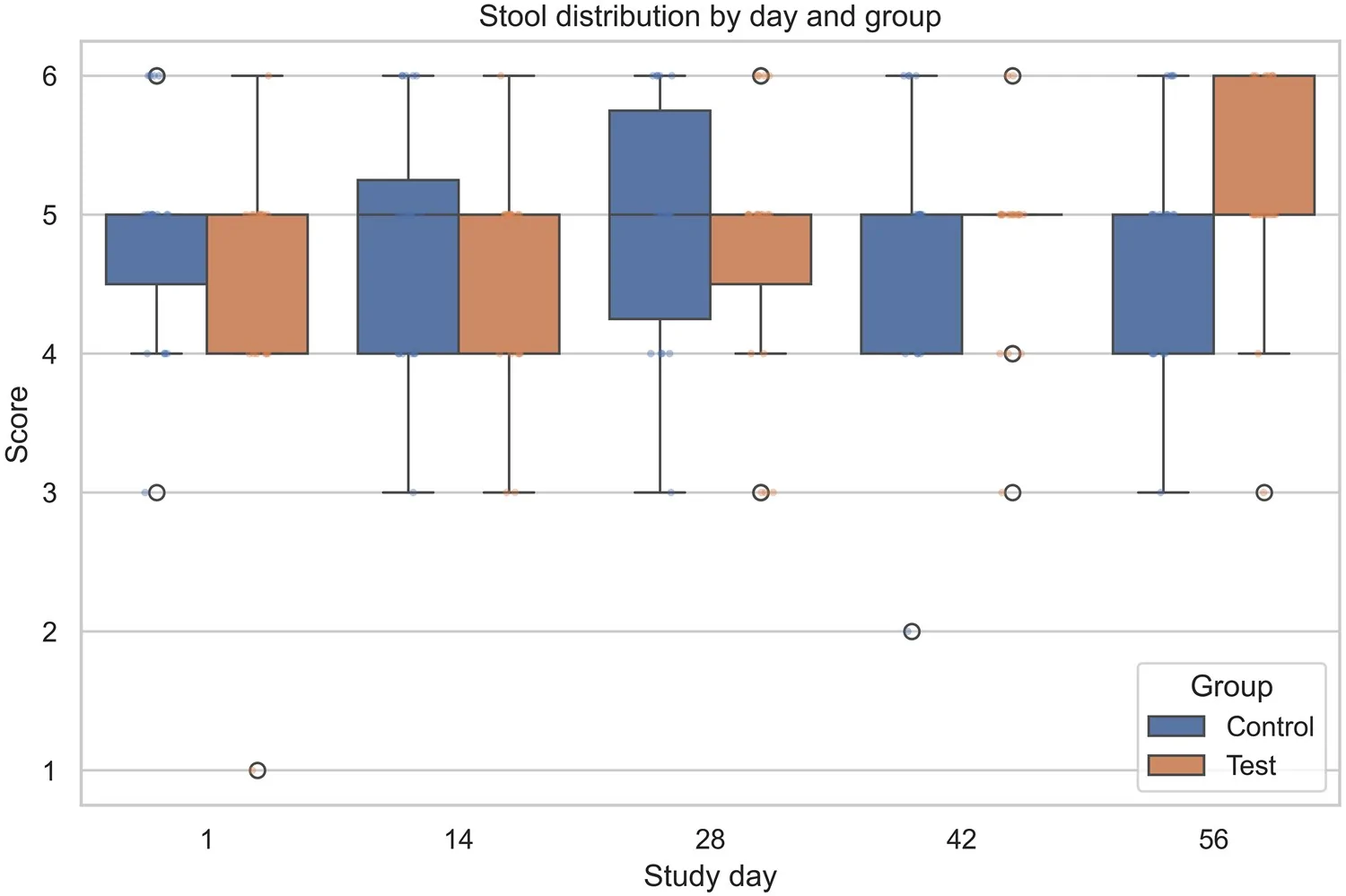
Stool scores were assessed by owners and measured on a scale of 1–6, where a grade 1 indicates liquid stool and a grade 6 indicates firm stools throughout the study duration for dogs fed the positive Control food or Test food.

Analysis of stool quality revealed no differences in stool scores between the Control and Test groups at d 1 (*P *= 1.00), 14 (*P *= 1.00), 28 (*P *= 1.00), 42 (*P *= 1.00), or 56 (*P *= 1.00); nor any changes over time for either group (Control: *P *= 1.00; Test: *P *= 1.00) (Table 9, Figure 4, and Table S1 and S2).

**Table 9:** Descriptive statistics for stool quality scores throughout the study for dogs fed the positive Control food and the Test food.

Group	Day	*n*	Median^1^	Q1	Q3	Min	Max
**Control**	1	19	5	4.5	5	3	6
**Control**	14	20	5	4	5.25	3	6
**Control**	28	18	5	4.25	5.75	3	6
**Control**	42	17	5	4	5	2	6
**Control**	56	19	5	4	5	3	6
**Test**	1	17	5	4	5	1	6
**Test**	14	18	5	4	5	3	6
**Test**	28	19	5	4.5	5	3	6
**Test**	42	18	5	5	5	3	6
**Test**	56	19	5	5	6	3	6

1 Stool quality scores were assessed by owners and measured on a scale of 1–6, with 1 being liquid diarrhea, 3 to 5 being normal, and 6 being firm.

### Coat quality

There was no difference between the Control or Test group’s coat quality at d 1 (*P *= 1.00), 28 (*P *= 1.00), or 56 (*P *= 1.00). Additionally, coat quality was maintained for both groups (Control: *P *= 0.908; Test: *P *= 1.00), when compared to baseline (Table 10 and Tables S1 and S2).

**Table 10:** Descriptive statistics of the total coat quality score throughout the study for dogs fed the positive Control food and the Test food.

Group	Day	*n*	Median^1^	Q1	Q3	Min	Max
**Control**	1	20	14	8.75	14	0	26
**Control**	28	20	11	8	14	0	26
**Control**	56	20	11.5	9.5	15.25	0	21
**Test**	1	18	12	10.25	15	0	23
**Test**	28	19	12	8.5	14	0	21
**Test**	56	19	12	9.5	13.5	0	19

1 Coat quality was assessed using a validated scoring system evaluating six features including alopecia, glossiness, greasiness, softness, scaliness and overall coat quality (Devriendt et al. 2021). A numerical value was assigned to categorize the absence or presence of each condition or its severity using a 5-point scale (1 = normal, 5 = poor). Total coat quality score was determined by calculating the sum of each coat quality question, with a score of 6 being the highest coat quality score achievable and a score of 30 being the lowest coat quality score achievable.

### Eating enthusiasm

Analysis of eating enthusiasm revealed no differences between the two groups on d 1 (*P *= 1.00), or 56 (*P *= 1.00) d 1 or 56. Furthermore, eating enthusiasm was maintained over time for both groups (Control: *P *= 0.427; Test: *P *= 0.215) (Table 11 and Tables S1 and S2).

**Table 11:** Descriptive statistics for eating enthusiasm throughout the study for dogs fed the positive Control food and the Test food.

Group	Day	*n*	Median^1^	Q1	Q3	Min	Max
**Control**	1	19	7	6	7	2	7
**Control**	56	19	6	5	7	1	7
**Test**	1	16	7	6	7	5	7
**Test**	56	19	6	4.5	7	1	7

1 Eating enthusiasm was evaluated by an emoji scale ranging from 1 (least enthusiasm to eat) through 7 (most enthusiasm to eat).

### Pet, owner, and treatment QoL

No differences were observed between the Control or Test groups for pet (*P *> 0.962), owner (*P *> 1.00), or treatment QoL (*P *> 1.00) at any point throughout the study. When evaluating these metrics across the study period, overall (Table S1), pet QoL scores did not change significantly for either the Control group (*P *= 0.309; medians at d 1, 28, and 56: 32.5, 35.0, and 37.5, respectively) or the Test group (*P *= 0.687; medians: 34.0, 37.0, and 41.0, respectively), though paired comparisons versus baseline indicated a significant localized improvement for the Control group at d 28 (*P *= 0.048). Similarly, treatment QoL remained consistent across the study period for both the Control group (*P *= 1.00; medians: 13.0, 12.0, and 12.0, respectively) and the Test group (*P *= 1.00; medians: 12.0, 14.0, and 13.0, respectively). Analysis of owner QoL revealed a significant overall effect during the study for the Test group (*P *= 0.022; medians: 47.0, 51.0, and 50.0, respectively), such that owner QoL improved (*P *= 0.029) by d 56 when compared to baseline (Table S2). Conversely, no significant differences over time were observed in the Control group’s owner QoL (*P *= 0.518; medians: 48.0, 51.0, and 46.5, respectively). Pet, owner, and treatment QoL are displayed in detail in Table 12.

**Table 12:** Descriptive statistics for quality of life throughout the study for dogs fed the positive Control food and the Test food.

Group	Day	*n*	Median^1^	Q1	Q3	Min	Max
**Owner QoL**							
** Control**	1	19	48	41	52.5	0	64
** Control**	28	17	51	45	56	32	63
** Control**	56	18	46.5	44.25	54	31	64
** Test**	1	18	47	39.25	55.75	0	59
** Test**	28	19	51	44	56.5	26	62
** Test**	56	17	50	44	60	29	62
**Pet QoL**							
** Control**	1	18	32.5	28.25	36.75	22	51
** Control**	28	17	35	33	40	16	50
** Control**	56	18	37.5	31.25	40	25	54
** Test**	1	17	34	30	44	17	51
** Test**	28	19	37	33	42.5	15	52
** Test**	56	17	41	32	45	18	52
**Treatment QoL**							
** Control**	1	19	13	12	14.5	7	19
** Control**	28	17	12	11	14	6	19
** Control**	56	18	12	10.25	13.75	7	21
** Test**	1	17	12	11	14	2	20
** Test**	28	19	14	12	16	9	17
** Test**	56	17	13	12	15	9	17

1 QoL was assessed by owners via three sets of questions focused on the pet, pet parents, and treatments on a scale of 0–4, with 4 being positive and 0 being negative.

## Discussion

This randomized double-blinded study demonstrated that both the positive Control (Hill’s Prescription Diet d/d Potato & Venison, Hill’s Pet Nutrition, Topeka, KS, USA) and Test (Hill’s Prescription Diet Derm Complete, Hill’s Pet Nutrition, Topeka, KS, USA) foods, when incorporated as an adjunctive therapy, produced statistically significant improvements in skin lesion severity (CADESI-4) over time. These results are consistent with prior reports of therapeutic dermatologic foods supporting skin barrier function and reducing inflammation in cAD (Glos et al. 2008; Witzel-Rollins et al. 2019; Watson et al. 2022, 2026). Lesion improvements likely reflect the foods’ provision of fatty acids and targeted nutrients that modulate cutaneous immune responses and support epidermal integrity (Drechsler et al. 2024). The benefit of polyunsaturated fatty acids integrated in dermatologic diets has been confirmed to reduce pruritus, reduce inflammatory responses, and improve coat quality in dogs with cAD (Glos et al. 2008; Schumann et al. 2014). It is likely that this mechanism played a role in the results of this study. Additionally, both foods include a singular uncommon protein source with no significant findings suggesting that one food outperformed the other. The venison and potato formulation used in the Control food and the egg and rice formulation in the Test food both displayed positive results over the 8-wk study period with owners noting that both diets were readily accepted by their pet. This is an important factor to emphasize as many pet owners struggle to employ a proper dietary trial or transition to a hypoallergenic food as a result of cost, palatability, or acceptance of the new diet by their pet.

Dogs were enrolled in the study in the Kansas City, KS area between the months of June through November. The study was designed to reduce the possibility of seasonal influence by enforcing the criteria of patients exhibiting non-seasonal pruritus. This was implemented to lessen the potential of seasonal influence during the study period. Additionally, this criterion was used to eliminate the possibility that dogs in the study could be evaluated outside of their most significant season, inadvertently making skin lesions, pruritus, or need for medications appear better independent of dietary modifications. The majority of dogs in the study were, however, evaluated during summer and fall when pollen levels are high within the environment. This highlights that the impacts of the foods were evaluated during an important time of year for many atopic patients.

Owner-reported PVAS showed no statistical improvements in either group over time. The interpretation of this data implies that dietary interventions may provide incomplete antipruritic effects that are observable within 8 wk. Furthermore, pruritus continues to be a challenging target when addressing cAD and related clinical signs. Improved outcomes may have been observed if the foods were employed beyond 2 mo as recognized in other studies with longer intervals evaluated (Watson et al. 2022).

Despite identifiable, positive improvements in skin lesions, pet QoL and QoL related to the pet’s treatment were unchanged throughout the study. This likely reflects fatigue most owners experience with ongoing management despite objective improvements with the skin. Owners often report poorer QoL measures in pets with advanced or chronic cAD, especially those that have elevated pruritus (Linek and Favrot 2010; Drechsler et al. 2024). However, increases in owner QoL were achieved on d 56 when compared to baseline for those owners with dogs fed the Test food.

It is imperative to recognize the complexity and overlap of cAD and CAFR to ensure that appropriate measures have been taken to distinguish the two in individual patients (Hensel et al. 2015; Nuttall et al. 2019). While historically viewed as fully separate entities, there is a strong consensus that CAFR and environmental AD can overlap. Because reactions to food components can present clinically as canine AD or serve as a flare factor in canine AD, dogs with CAFR may be indistinguishable clinically from canine AD (Hensel et al. 2015). The International Task Force on cAD also supports the concept that food components trigger flares in hypersensitive dogs (Olivry et al. 2010). Furthermore, adverse food reactions concurrent to AD have been documented to account for 20% to 30% or more of dogs (Bhagat et al. 2017). As such, a large percentage of patients suffer from environmental-induced AD, a smaller subset is impacted by both, and an even smaller portion is controlled by food alone, it is highly clinically relevant to include both populations when evaluating therapies intended for a real-world setting. Regarding the two dogs with confirmed food allergies: both patients were already being appropriately managed on hydrolyzed diets (one on a hydrolyzed chicken food and the other on a hydrolyzed soy food) prior to their enrollment. Consequently, their food allergies were controlled at baseline, and we can infer they had not been previously exposed to an allergenic dietary protein before transitioning to the study foods.

In the present study, a strict elimination food trial was not required for enrollment; rather, the inclusion criteria were intentionally designed to reflect a real-world population where these conditions frequently overlap. To proactively address this overlap, both the Control and Test foods utilize uncommon protein sources. It should be noted that positive results in skin lesions may not have the same repeatability if dogs are part of the smaller percentage of pets that are impacted by egg or venison protein sources (Leistra et al. 2001). Furthermore, the Control food is formulated with limited ingredients and nutrients to support the skin and could be considered for elimination diet trials for adverse food reactions (Fritsch et al. 2010; Weemhoff et al. 2021), while the Test food addresses both conditions simultaneously by combining an uncommon protein with immunomodulating components.

Both foods were well tolerated, with adverse events similar to those reported with other hypoallergenic formulas used in veterinary dermatology clinics (Roudebush and Cowell 1992; Valentine 2020; Tinsley et al. 2024). Of the reported adverse events, the most relevant is the relapse in pyoderma, which occurred on d 56 for one patient fed the Control food. This is particularly important to recognize since staphylococcal infections can negatively stimulate pruritus and lead to CADESI-4 exacerbations (Santoro et al. 2015; Santoro and Hoffmann 2016). This mechanism of action takes place as a result of Staphylococcal superantigens, which can amplify allergic inflammation and mast cell responses (Santoro et al. 2015; Santoro and Hoffmann 2016). This highlights yet another challenge faced in managing patients with cAD as skin barrier dysfunction plays a significant role (Nuttall et al. 2019; Drechsler et al. 2024; Banovic 2025; Marsella 2026). This also stresses the importance of infection control in cAD trials and may have contributed to variability in subjective outcomes in this study.

A primary limitation of this study is the absence of a true negative control group. A true negative control group, such as maintaining dogs on a non-therapeutic habitual food, was not utilized in this study due to ethical considerations regarding the active disease burden of the enrolled dogs. Placing these dogs with a high disease burden on a sub-optimal food while potentially withholding stabilizing medications would risk an unacceptable decline in their QoL. Therefore, this study was designed to compare two distinct, therapeutic nutritional strategies to evaluate their comparative efficacy as adjunctive therapies in a real-world setting. Another limitation of the study includes the relatively short duration of evaluation. An 8-wk study period was chosen based on the timeframe that is commonly recommended to perform an elimination diet trial for pets with suspected food-induced AD. A longer study duration, however, may have shown differences over time in some of the endpoints that did not change when compared to baseline. Additionally, patients varied significantly in their medication requirements. While this medication variability introduces potential confounding factors, a strict inclusion criterion requiring dermatology-related medications to remain unchanged for at least 1 mo prior to enrollment helped mitigate this. Ultimately, this variability accurately reflects the real-world clinical presentation and multimodal management of cAD. All dogs enrolled in the study were patients seen at a specialty dermatology clinic and embodied a population of cAD with greater disease burden compared to the general population. Additionally, patients varied significantly in their medication requirements. A more homogeneous set of patients could have been considered for enrollment with either uniform needs or no medications administered throughout the study period. Due to the expected decline in patient’s QoL with medication withdrawal, this type of standardization was not made a requirement for ethical reasons. Another limitation of this study is the influence of prior diets that were fed before enrollment. A detailed record of all kibble or food items enrolled pets was receiving prior to enrollment were not exclusively evaluated. This is important to consider as we cannot entirely exclude the fact that this could have influenced early CADESI-4 drops as lesser than or equivocal food quality could have been administered previously. Moreover, there could have been an abrupt allergen avoidance upon starting the Control or Test food as an elimination diet trial was not required for enrollment. It is possible that food-induced cAD could have played a larger role in the outcomes of this study. Confirmation of this could have been observed upon reintroduction of previous diets, which is recognized as another limitation of this study. Ultimately, diversity in cAD phenotype, concurrent therapies, and individual immune responses allowed for variability in this study. This, unfortunately, is an unavoidable challenge in a real-world multimodal study that must prioritize patient QoL and concurrent active disease.

Despite these limitations, the results provide valuable data on two commonly recommended veterinary dermatologic foods. While we initially hypothesized that the higher concentrations of Omega-3 fatty acids and inclusion of flavonoid-containing ingredients in the Test food might drive superior clinical outcomes, no significant differences were observed between the Control and Test groups for any of the endpoints assessed. Importantly, this lack of superiority does not diminish the efficacy of the food. Instead, it highlights a positive clinical finding that both the established, positive Control food and Test food perform well as adjunctive therapies. Both foods successfully facilitated significant, independent improvements over time, demonstrating that either nutritional approach is an effective tool for multimodal management of cAD. Specifically, when used as part of a multimodal approach to cAD, both foods significantly reduced skin lesions and maintained concurrent medication needs. These findings support dietary modification as a useful adjunctive strategy in veterinary dermatology. This emphasizes the need for larger and longer studies evaluating diet-specific mechanisms and their role in allergic skin diseases.

## Conclusion

The positive Control, Hill’s Prescription Diet d/d Potato & Venison (Hill’s Pet Nutrition, Topeka, KS, USA), and Test, Hill’s Prescription Diet Derm Complete Adult (Hill’s Pet Nutrition, Topeka, KS, USA), foods utilized in the present study both provided benefits to patients with cAD. This study demonstrated improvements in CADESI-4 scores over time in both Control and Test groups and maintenance of medication scores over an 8-wk period. Furthermore, the owners of dogs in the Test group reported improved QoL over time. Additional studies are required to evaluate the added benefits over a longer period of time. These results could help guide more targeted nutritional recommendations in the dermatologic setting.

## Supplementary Material

skag251_Supplementary_Data

## Data Availability

The raw data supporting the conclusions of this article will be made available by the authors upon reasonable request.

## Abbreviations

ASIT: allergen specific immunotherapy
cAD: canine atopic dermatitis
CADESI-4: canine atopic dermatitis and severity index, 4th iteration
CAFR: cutaneous adverse food reaction
DHA: docosahexaenoic acid
EPA: eicosapentaenoic acid
GI: gastrointestinal
ME: metabolizable energy
NFE: nitrogen-free extract
PVAS: Pruritus Visual Analog Scale
QoL: quality of life
