# Supplementary material for: A randomized, double-blinded trial to evaluate two therapeutic foods as adjunctive therapies in dogs with environmental and/or food-induced atopic dermatitis
Source: J Anim Sci. 2026 Aug 11;104:skag251. doi: 10.1093/jas/skag251 (PMC13588571; doi:10.1093/jas/skag251)
Supplement: Supplementary file 1 [file skag251_supplementary_data.docx]

**Supplementary Table 1.** Pairwise Comparisons vs. Baseline: Wilcoxon Signed-Rank Test.

Each follow-up time point was compared to baseline within each group using the Wilcoxon signed-rank test. A negative rank-biserial correlation indicates that scores decreased (i.e., improved for disease severity endpoints) relative to baseline. A **bolded** value indicates *P* ≤ 0.05 compared to baseline.

| **Endpoint** | **Group** | **Comparison** | **N** | **Wilcoxon W** | **p (raw)** | **p (Holm)** | **Rank-biserial r** |
| --- | --- | --- | --- | --- | --- | --- | --- |
| CADESI-4 | Control | D14 vs D-7 | 20 | 1.5 | **< 0.001** | **0.001** | -0.895 |
| CADESI-4 | Control | D28 vs D-7 | 20 | 0 | **< 0.001** | **< 0.001** | -1 |
| CADESI-4 | Control | D56 vs D-7 | 20 | 0 | **< 0.001** | **< 0.001** | -1 |
| CADESI-4 | Test | D14 vs D-7 | 19 | 38 | 0.02 | 0.102 | -0.684 |
| CADESI-4 | Test | D28 vs D-7 | 17 | 8 | **<0.001** | **0.002** | -0.765 |
| CADESI-4 | Test | D56 vs D-7 | 19 | 0 | **< 0.001** | **< 0.001** | -1 |
| Total coat quality score | Control | D28 vs D1 | 20 | 25 | 0.045 | 0.406 | -0.467 |
| Total coat quality score | Control | D56 vs D1 | 20 | 67 | 0.257 | 1 | -0.368 |
| Total coat quality score | Test | D28 vs D1 | 18 | 73 | 0.609 | 1 | -0.111 |
| Total coat quality score | Test | D56 vs D1 | 18 | 57.5 | 0.583 | 1 | 0 |
| Eating enthusiasm | Control | D56 vs D1 | 18 | 10.5 | 0.142 | 0.427 | -0.333 |
| Eating enthusiasm | Test | D56 vs D1 | 16 | 3 | 0.054 | 0.215 | -0.714 |
| Medication score | Control | D28 vs D-7 | 19 | 5 | 0.121 | 0.356 | -0.429 |
| Medication score | Control | D56 vs D-7 | 19 | 2 | 0.071 | 0.304 | -0.667 |
| Medication score | Test | D28 vs D-7 | 18 | 3 | 0.031 | 0.217 | -0.75 |
| Medication score | Test | D56 vs D-7 | 19 | 12.5 | 0.212 | 0.356 | -0.556 |
| Owner QoL | Control | D28 vs D1 | 16 | 17 | 0.014 | 0.086 | 0.467 |
| Owner QoL | Control | D56 vs D1 | 18 | 55 | 0.307 | 1 | 0.176 |
| Owner QoL | Test | D28 vs D1 | 18 | 17 | 0.008 | 0.057 | 0.75 |
| Owner QoL | Test | D56 vs D1 | 17 | 9 | **0.004** | **0.029** | 0.733 |
| Pet QoL | Control | D28 vs D1 | 15 | 13 | **0.005** | **0.048** | 0.6 |
| Pet QoL | Control | D56 vs D1 | 18 | 30.5 | 0.029 | 0.232 | 0.294 |
| Pet QoL | Test | D28 vs D1 | 17 | 48 | 0.777 | 1 | 0 |
| Pet QoL | Test | D56 vs D1 | 17 | 29.5 | 0.046 | 0.309 | 0.375 |
| Treatment QoL | Control | D28 vs D1 | 16 | 42.5 | 0.828 | 1 | -0.077 |
| Treatment QoL | Control | D56 vs D1 | 18 | 40 | 0.43 | 1 | -0.286 |
| Treatment QoL | Test | D28 vs D1 | 17 | 27.5 | 0.206 | 1 | 0.385 |
| Treatment QoL | Test | D56 vs D1 | 17 | 33.5 | 0.394 | 1 | 0.385 |
| PVAS | Control | D14 vs D-7 | 20 | 39 | 0.07 | 0.912 | -0.412 |
| PVAS | Control | D28 vs D-7 | 19 | 30.5 | 0.049 | 0.685 | -0.375 |
| PVAS | Control | D42 vs D-7 | 18 | 41.5 | 0.485 | 1 | -0.143 |
| PVAS | Control | D56 vs D-7 | 20 | 33 | 0.019 | 0.291 | -0.333 |
| PVAS | Test | D14 vs D-7 | 19 | 75 | 0.645 | 1 | -0.222 |
| PVAS | Test | D28 vs D-7 | 19 | 50 | 0.564 | 1 | -0.067 |
| PVAS | Test | D42 vs D-7 | 18 | 44 | 0.914 | 1 | -0.077 |
| PVAS | Test | D56 vs D-7 | 19 | 50.5 | 0.899 | 1 | 0.143 |
| Stool score | Control | D14 vs D1 | 19 | 22.5 | 1 | 1 | 0.111 |
| Stool score | Control | D28 vs D1 | 17 | 12 | 0.705 | 1 | 0.143 |
| Stool score | Control | D42 vs D1 | 16 | 15 | 0.66 | 1 | 0.25 |
| Stool score | Control | D56 vs D1 | 18 | 27.5 | 1 | 1 | 0 |
| Stool score | Test | D14 vs D1 | 17 | 25 | 0.782 | 1 | 0 |
| Stool score | Test | D28 vs D1 | 17 | 25 | 0.449 | 1 | 0.273 |
| Stool score | Test | D42 vs D1 | 16 | 6 | 0.317 | 1 | 0.333 |
| Stool score | Test | D56 vs D1 | 17 | 2.5 | 0.047 | 0.71 | 0.714 |

**Supplementary Table 2.** Global Trend: Friedman Test.

The Friedman test assesses whether scores differ significantly across all available time points within a group. A **bolded** value indicates *P* ≤ 0.05 and a non-flat trajectory.

| **Endpoint** | **Group** | **Time points** | **N** | **Friedman chi2** | **p (raw)** | **p (Holm)** |
| --- | --- | --- | --- | --- | --- | --- |
| CADESI-4 | Control | Friedman across D-7,D14,D28,D56 | 20 | 42.66 | **< 0.001** | **< 0.001** |
| CADESI-4 | Test | Friedman across D-7,D14,D28,D56 | 17 | 28.49 | **< 0.001** | **< 0.001** |
| Total coat quality score | Control | Friedman across D1,D28,D56 | 20 | 4.35 | 0.113 | 0.908 |
| Total coat quality score | Test | Friedman across D1,D28,D56 | 18 | 0.58 | 0.747 | 1.000 |
| Medication score | Control | Friedman across D-7,D28,D56 | 19 | 4.26 | 0.119 | 0.356 |
| Medication score | Test | Friedman across D-7,D28,D56 | 18 | 5.6 | 0.061 | 0.304 |
| Owner QoL | Control | Friedman across D1,D28,D56 | 15 | 4.54 | 0.061 | 0.304 |
| Owner QoL | Test | Friedman across D1,D28,D56 | 17 | 12.03 | **0.002** | **0.022** |
| Pet QoL | Control | Friedman across D1,D28,D56 | 15 | 6.24 | 0.044 | 0.309 |
| Pet QoL | Test | Friedman across D1,D28,D56 | 17 | 3.97 | 0.137 | 0.309 |
| Treatment QoL | Control | Friedman across D1,D28,D56 | 15 | 1.04 | 0.594 | 1.000 |
| Treatment QoL | Test | Friedman across D1,D28,D56 | 17 | 3.16 | 0.594 | 1.000 |
| PVAS | Control | Friedman across D-7,D14,D28,D42,D56 | 17 | 3.16 | 0.386 | 0.386 |
| PVAS | Test | Friedman across D-7,D14,D28,D42,D56 | 18 | 1.77 | 0.778 | 0.778 |
| Stool score | Control | Friedman across D1,D14,D28,D42,D56 | 14 | 0.51 | 0.972 | 0.972 |
| Stool score | Test | Friedman across D1,D14,D28,D42,D56 | 16 | 6.29 | 0.179 | 0.179 |

**Supplementary Figure 1.** Canine stool quality grading chart.

**What is the typical consistency of your dog’s stool in the past week?**
